# Randomized study evaluating the efficacy of sustained-release dexamethasone with or without prompt laser for branch retinal vein occlusion-related macular edema

**DOI:** 10.22336/rjo.2025.60

**Published:** 2025

**Authors:** Ashish Markan, Shorya Vardhan Azad, Brijesh Takkar, Rohan Chawla

**Affiliations:** 1 Dr. Rajendra Prasad Centre for Ophthalmic Sciences, AIIMS, New Delhi, India; 2 L V Prasad Eye Institute, Hyderabad, India

**Keywords:** RCT, BRVO, macular edema, Dexamethasone implant, laser therapy, BCVA = Best-corrected visual acuity, BRVO = Branch retinal vein occlusion, CMT = Central macular thickness, CS = Contrast sensitivity, CV = Color vision, DI = Dexamethasone implant, FA = Fluorescein angiography, IOP = Intraocular pressure, IVB = Intravitreal bevacizumab, IVR = Intravitreal ranibizumab, ME = Macular edema, OCT = Optical coherence tomography, PRN = Pro re nata (as needed), RVO = Retinal vein occlusion, TA = Triamcinolone acetonide, VEGF = Vascular endothelial growth factor

## Abstract

**Purpose:**

To evaluate the combination therapy of sustained-release dexamethasone implant (DI) with/without prompt laser in patients with macular edema secondary to BRVO.

**Methods:**

Forty eyes of 40 patients with BRVO were randomized into two groups. Group 1 received a single dose of intravitreal injection of DI, and Group 2 received a single dose of DI followed by prompt laser treatment on the 7th day. Primarily, outcome measures noted at 3, 6, 9, and 12 months’ follow-up were improvement in best-corrected visual acuity (BCVA) and central macular thickness (CMT). Secondary outcome measures were change in intraocular pressure (IOP), color vision (CV), and contrast sensitivity (CS).

**Results:**

Mean age of patients in Groups 1 and 2 was 60.30 + 11.59 years and 52.30 + 10.86, respectively. Mean BCVA (logMAR units) in Groups 1 vs. 2 was 0.72 vs. 0.67 at baseline (p=0.286), 0.38 vs. 0.34 at 3 months (p=0.99), 0.29 vs. 0.36 at 6 months (p=0.006), 0.23 vs. 0.36 at 9 months (p=0.001), and 0.19 vs. 0.38 at 12 months (p=0.001), respectively. Mean CMT (μ) in Groups 1 and 2 was 519.5 and 491.1 at baseline (p=0.33), 285.8 and 334.2 at 3 months (p=0.035), 343.1 and 328.9 at 6 months (p=0.294), 309.6 and 328.7 at 9 months (p=0.009), and 283.4 and 316.42 at 12 months (p=0.231), respectively. CS and CV were significantly better in Group 1 as compared to Group 2 at 12 months, while IOP was similar.

**Discussion:**

Our study demonstrated that prompt addition of laser therapy to sustained-release dexamethasone implant did not confer additional benefit in BRVO-related macular edema and might, in fact, compromise long-term visual outcomes. While both groups showed comparable early improvement, monotherapy with dexamethasone resulted in superior BCVA, color vision, and contrast sensitivity at 12 months. These findings suggested that concurrent use of steroids and prompt laser might interfere with retinal healing. Differences in outcomes compared to earlier studies might relate to the timing of laser application, as deferred laser appears to be more effective. Larger trials with longer follow-up are warranted to confirm these observations.

**Conclusion:**

Combination therapy of laser with intravitreal sustained-release DI leads to poorer visual outcomes and may indicate a negative impact of steroid therapy on healing.

## Introduction

Branch retinal vein occlusion (BRVO) is often complicated by macular edema in its natural course, resulting in moderate vision loss. The pathogenesis is believed to be multifactorial. Once the retinal vein gets occluded, an increase in hydrostatic pressure leads to leakage of fluid across the vessel wall, resulting in local edema [1]. Subsequently, upregulation of inflammatory mediators and low-grade chronic inflammation is induced by localized endothelial damage and ischemia at the site of obstruction. These mediators include prostaglandins, leukotrienes, ICAM-1, TNF-α, integrins, and vascular endothelial growth factor (VEGF) [2-5]. A vicious cycle may be set up between ischemia and such mediators, sometimes resulting in chronic and sustained macular edema [6].

Monotherapy is generally recommended as the standard of care for BRVO-related macular edema. Although lasers had been the gold standard for treatment earlier, many other modalities of treatment have been extensively explored lately. Recent studies suggest that anti-VEGF agents deliver superior results compared to laser. However, multiple injections raise concerns about the risks associated with intravitreal injections as well as their financial viability in developing nations. On the other hand, steroids have been effective, but concerns of ocular hypertension and cataract have limited their use [7,8].

With recent advocacy of combination therapy, it is postulated that combining intravitreal steroid therapy with laser photocoagulation may maximize visual outcomes as steroids decrease macular edema, thereby increasing laser uptake, as also observed with the combination of anti-VEGF with laser. Also, combination therapy may require fewer injections while being non-inferior to anti-VEGF therapy [9]. Some studies have shown that combined treatment is synergistic in increasing visual acuity and lengthening the time between injections, as compared to monotherapy with dexamethasone implant or laser alone [10].

The current study compares the efficacy of the combination therapy of sustained-release dexamethasone and prompt laser with steroid monotherapy in BRVO-related macular edema. We also evaluated other qualitative visual functions, like color vision and contrast sensitivity, and the rise in intraocular pressure secondarily.

## Methods

This is a randomized, prospective, comparative study performed at a tertiary eye care facility in northern India over 12 months. The study adhered to the tenets of the Declaration of Helsinki, and institutional review board ethical clearance was obtained (Dr. Rajendra Prasad Centre for Ophthalmic Sciences, AIIMS, New Delhi, India) (IEC/NP-362/11, RP-05/12). Individual informed consent was also taken for all the procedures involved.

Consecutive patients with treatment-naive macular edema secondary to BRVO, consenting to participate in the study, were included. Only patients having baseline Snellen’s best corrected visual acuity (BCVA) worse than 6/12 for at least 6 weeks and central macular thickness (CMT) > 250 microns on optical coherence tomography (OCT) (Carl Zeiss Cirrus OCT) were treated. Patients with other ocular disorders (including glaucoma and glaucoma suspects) and a history of intraocular surgery other than cataract surgery were excluded from the study. All these patients underwent a meticulous ophthalmic examination, including BCVA, IOP, slit lamp biomicroscopy, and dilated fundus examination with indirect ophthalmoscopy. Fluorescein Angiography (FA) was performed in all patients, and those with macular ischemia were excluded.

Forty eyes of 40 patients satisfying the inclusion-exclusion criteria were included in the study and randomized into two groups of 20 eyes each. Group 1 received a single dose of intravitreal injection of sustained-release dexamethasone implant (700 microgram). In comparison, Group 2 received a single dose of intravitreal injection of the dexamethasone implant (700 microgram) followed by grid laser treatment on the seventh day post-injection. Laser photocoagulation was performed with a spot size of 50 microns and an exposure time of 0.1 seconds. The power was adjusted and started at 50 MW and increased in steps of 10 MW to produce mild intensity covering areas of capillary leakage as seen on FFA, one burn width apart. All patients were allowed retreatment with repeat dexamethasone implant if there was a fall in BCVA (> 2 lines) or an increase in CMT (>100 microns). BCVA and CMT were the primary outcome measures. These were recorded at 3, 6, 9, and 12 months. Secondary outcome measures were a rise in IOP, changes in color vision, and contrast sensitivity.

Data was entered into Microsoft Excel spreadsheets, and analysis was done using SPSS software (Version 16). One-way Analysis of variance (ANOVA) was used to compare the baseline data of the two groups. Pearson’s Chi-square test was used for analyzing non-parametric variables. Spearman’s rank analysis was applied to assess the correlation between continuous variables. Only a 2-tailed p-value of less than 0.05 was considered statistically significant.

## Results

A total of 40 eyes of 40 patients satisfying the inclusion-exclusion criteria were included in the study, comprising 17 males and 23 females. The mean age of patients in Group 1 was 60.30 ± 11.59 years, while in Group 2 it was 52.30 ± 10.86 years. Patient demographics (age, gender, associated systemic diseases) and baseline ocular characteristics were comparable across both groups.

Overall, 24/40 eyes received a single dose of dexamethasone implant, whereas 14 eyes required repeat injections. Seven patients required repeat injection in each group following a mean interval of 2.95 + 4.548 months and 2.90 + 4.400 months in groups 1 and 2, respectively.

**Fig. 1:**
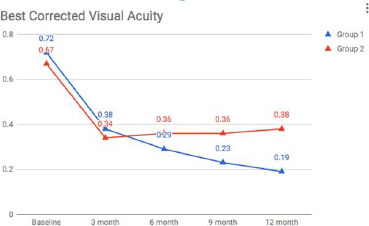
Change in the BCVA over a period of 12 months from baseline in group 1 and group 2

**Fig. 2:**
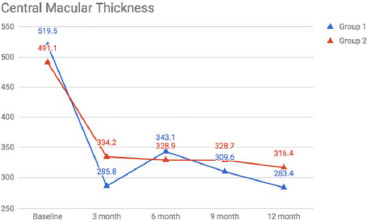
Change in the central macular thickness over a period of 12 months from baseline in group 1 and group 2

Mean BCVA (LogMAR units) in Groups 1 vs. 2 was 0.72 + 0.34 vs. 0.67 + 0.29 at baseline (p=0.286), 0.38 + 0.33 vs. 0.34 + 0.29 at 3 months (p=0.99), 0.29 + 0.19 vs. 0.36 + 0.36 at 6 months (p=0.006), 0.23 + 0.14 vs. 0.36 + 0.38 at 9 months (0.001) and 0.19 + 0.10 vs. 0.38 + 0.38 at 12 months (p= 0.001) respectively (**Fig. 1**) (**Table 1**). Mean CMT (u) in Group 1 and 2 was 519.5 + 146 and 491.1 + 167 at baseline (p=0.33), 285.8 + 80 and 334.2 + 137 at 3 months (p=0.035), 343.1 + 99 and 328.9 + 134 at 6 months (p=0.294), 309.6 + 80 and 328.7 + 131 at 9 months (p=0.009) and 283.4 + 102 and 316.42 + 140 at 12 months (p=0.231) respectively (**Fig. 2**) (**Table 1**).

**Table 1:** Comparison of best-corrected visual acuity (BCVA), central macular thickness (CMT), and intraocular pressure (IOP) between Group 1 (DI) and Group 2 (DI + Laser)

	BASELINE			3 MONTHS			6 MONTHS			9 MONTHS			12 MONTHS		
	Group 1	Group 2	P value	Group 1	Group 2	P value	Group 1	Group 2	P value	Group 1	Group 2	P value	Group 1	Group 2	P value
BCVA (logMAR)	0.72 ± 0.34	0.67 ± 0.29	0.28	0.38 ± 0.33	0.34 ± 0.29	0.99	0.29 ± 0.19	0.36 ± 0.36	0.06	0.23 ± 0.14	0.36 ± 0.38	0.00	0.19 ± 0.10	0.38 ± 0.38	0.00
CMT (µm)	519.5 ± 146	491.1 ± 167	0.33	285.8 ± 80	334.2 ± 137	0.03	343.1 ± 99	328.9 ± 134	0.29	309.6 ± 80	328.7 ± 131	0.00	283.4 ± 102	316.4 ± 140	0.23
IOP (mmHg)	12.4 ± 2.5	14.0 ± 2.1	0.42	14.7 ± 5.2	14.0 ± 2.5	0.08	13.1 ± 2.0	13.0 ± 2.1	0.61	13.2 ± 3.1	14.1 ± 2.4	0.33	14.0 ± 4.0	14.5 ± 3.5	0.80

Contrast sensitivity and color vision were significantly better in Group 1 as compared to Group 2 at 12 months (p<0.05) (**Table 2**). Rise of intraocular pressure (>25 mm Hg) was noted in only one patient in group 1, who was managed on topical medications. No other serious ocular adverse effects were observed in any patient throughout the study period.

**Table 2:** Comparison of color vision (CV) and contrast sensitivity (CS) between Group 1 (DI) and Group 2 (DI + Laser)

	BASELINE			3 MONTHS			6 MONTHS			9 MONTHS			12 MONTHS		
	Group 1	Group 2	P value	Group 1	Group 2	P value	Group 1	Group 2	P value	Group 1	Group 2	P value	Group 1	Group 2	P value
CV (units)	1.11 ± 0.49	1.19 ± 0.50	0.97	1.44 ± 0.37	1.40 ± 0.46	0.92	1.58 ± 0.11	1.31 ± 0.54	0.00	1.50 ± 0.32	1.38 ± 0.42	0.01	1.55 ± 0.38	1.38 ± 0.46	0.00
CS	8.0 ± 7.9	8.8 ± 8.0	0.80	12.8 ± 7.2	12.1 ± 7.1	0.56	16.4 ± 2.0	11.6 ± 7.1	0.00	15.5 ± 4.5	13.2 ± 6.7	0.35	16.2 ± 2.1	12.6 ± 6.7	0.04

## Discussion

Modern-day medicine has made several treatment options available to us for the same disease, albeit acting through different pathways. Recently, there has been a change in thinking, exploring the viability of combining different therapies to maximize functional outcomes. Still, combining one approach may affect the other’s mode of action, resulting in variable outcomes. Hence, the purpose of our study was not only to evaluate the efficacy of the combination therapy of dexamethasone implant with Laser, but also to evaluate the impact of one therapy over the other.

Earlier studies have shown that combination therapy can provide better improvement in both BCVA and CMT, thereby minimizing the number of injections and increasing the duration of effects in patients having BRVO [10]. This has been advocated as synergism. On comparing BCVA of the 2 study groups, we found that there was no significant difference for the first 6 months. Still, the BCVA was significantly better at 9 and 12 months in Group 1 as compared to Group 2, showing that the addition of prompt laser to dexamethasone implant has detrimental effects on the visual functions in the long term. Further, there was no difference in terms of need or duration for retreatment between the 2 study groups. This contrasts with a study done by Pichi et al. [10], who have shown that the combination of dexamethasone implant and macular grid laser is synergistic in increasing final BCVA and lengthening the time between injections. The difference between the results can be attributed to two reasons. Firstly, our study had a longer follow-up as compared to Pichi et al., who evaluated the results at 6 months of follow-up. Secondly, our study was designed to administer laser treatment at 1 week post-injection, whereas in the study by Pichi et al., the laser was deferred until 6-8 weeks. Combination therapy may work best with deferred laser when the effect of the long-acting steroid begins to wane off [11]. It is known that the maximum release of dexamethasone is in the first 2 months post-injection with a steady decrease thereafter, thereby justifying the possible efficacy of deferred laser [12].

Also, our results showed that combination therapy significantly affects at least two qualitative aspects of vision - the color vision and contrast sensitivity. Experimental studies have shown that the laser scar decreases over time due to migration of photoreceptors from the untreated surrounding areas, filling in the damaged outer retina. Healthy adjacent photoreceptors have been shown to repopulate regions of photoreceptor damage in a process associated with actin [13]. A Study by Nomoto et al. [14] evaluated the effect of intravitreal triamcinolone acetonide (TA) on the healing of retinal photocoagulation lesions using drug and laser dosing typically employed in clinical practice. In their study, 40 Dutch belted rabbits underwent laser treatment following intravitreal injections of either 2 mg TA/50 μL or balanced salt solution either 1 week before or immediately after treatment. Lesion grades were assessed acutely ophthalmoscopically and by a masked observer histologically at 1, 3, 7, 30, and 60 days. They concluded that intravitreal injection of steroid affects the healing of laser retinal scars by modifying the migration of photoreceptors and activation of Muller cells, processes essential for the healing of laser scars. Consequently, giving an intravitreal steroid injection previously or concurrently with photocoagulation significantly interferes with the healing of the lesions, thereby leaving wider residual scarring. This phenomenon can account for decreased contrast sensitivity and color vision in the combination group as compared to the group where only the steroid was injected. This also explains why a significant difference was observed amongst the groups in terms of final visual acuity and not in the initial periods, when the process of photoreceptor migration would not have been predominant.

Limitations of our study are the absence of a deferred laser combination and the inclusion of only laser groups, which could have further helped in explaining the effects of steroids on laser and vice versa. For the same reason, we could not comparatively study the evolution of laser scars throughout follow-up. However, poorer outcomes in group 1 for the primary as well as secondary outcome measures indicate prompt laser in conjunction with steroids to be an inferior form of combination therapy. These results should be further validated with larger studies having multiple groups, as mentioned earlier. Further, studying the outer retinal layer from the perspective of combination vs. monotherapy therapy may also help.

Comparison with other combination strategies involving laser for BRVO-related macular edema is presented in **Table 3**.

**Table 3:** Comparison between combination therapies involving laser for BRVO-related ME

Study	Design, year, place, sample size	Method of combination	Conclusion	Comments
Current Study	Randomized, North India, 40 patients.	Sustained-release steroid with or without prompt laser	No initial benefit, poor outcomes at 1 year	Laser was done on the 7th day post-injection.
Pichi et al. [10]	Randomized, Italy, 50 patients.	Sustained-release steroid or without deferred laser	The combination of Ozurdex implant and macular grid laser is synergistic in increasing BCVA and lengthening the time between injections.	Laser was done 6-8 weeks post-injection.
Tomomatsu et al. [1]	Randomized, Japan, 38 patients.	IVB with or without laser	Vision significantly improved in the combination group at 6 months, but not in the other group. Less recurrence of ME	Targeted laser done initially on more than five disc diameters of ischemia
Campochiaro et al. [2]	Randomized, USA, 42 patients.	IVR with or without laser	No long-term benefit of laser in terms of vision, resolution of ME, or requirement of injections	Scatter and macular grid laser done after 6-monthly IVR in PRN phase
Wykoff et al. [3]	Randomized, USA, 2016, 30 patients.	IVR with or without laser	No appreciable impact on visual outcomes or treatment burden	Peripheral targeted laser for recalcitrant ME due to ischemic RVO
Pielen et al. [4]	Randomized, Germany, 30 patients.	IVR vs. laser vs. IVR+ laser	No enhancement of outcomes or benefit on recurrence rate at 6 months	Laser and injection on the first day itself in the combination group
Azad et al. [9]	Randomized, India, 30 patients.	IVR + Laser vs. IVB + Laser vs. Laser	Both ranibizumab and bevacizumab combined with laser photocoagulation, resulted in better outcomes than grid laser treatment.	A laser was done on the 7th day post-injection
Adelman et al. [5]	Non-randomized, Europe- 29 countries, online questionnaire-based, 380 cases of BRVO **#**	All therapies, including combination and surgical treatments, were evaluated.	The addition of grid laser or steroid to anti-VEGF therapy did not improve visual results in comparison to anti-VEGF alone	Suggested randomized trials for accurate conclusions

BRVO = Branch retinal vein occlusion, ME = macular edema, IVB = Intravitreal bevacizumab, IVR = Intravitreal ranibizumab, PRN = pro re nata,

The trial included CRVO patients. An extensive multicentre study included both CRVO and BRVO and found vitrectomy to be superior to medical therapy.

Although racial differences, parameters/type of laser, and timing/type of combination therapy may lead to contrasting results across studies, it is evident that most of the studies do not show any beneficial effect. However, most of these studies have been done using anti-VEGF agents (ranibizumab/bevacizumab), and the literature lacks information regarding the nature of the interaction between sustained-release steroid and laser in counteracting BRVO-related ME. There are reports of using steroids in combination with anti-VEGF, yielding superior outcomes than anti-VEGF alone in terms of visual gain, reduction of CMT, and longer duration of action [15-18]. It should be noted that modern laser technologies based on targeted laser (including BRVO cases) are more controlled and effective in tackling the “dry retina” [19]. Hence, the timing of combination therapy, prompt or deferred, must be evaluated carefully before considering it as inferior or ineffective to the standard of care [20].

## Conclusion

To summarize, the addition of prompt macular laser to intravitreal sustained-release steroid injection leads to poorer visual outcomes in our subset of patients. These differences were more evident late in follow-up. These results indicate a possible negative interaction between steroid therapy and the healing process following laser, and should be evaluated further.
